# Coeliac trunk origin of bilateral inferior phrenic arteries

**DOI:** 10.3389/fsurg.2026.1697766

**Published:** 2026-03-23

**Authors:** Raghad Abdulaziz Almansour, Sumar Chan, Jun Mun Teoh, Rasyidah Rehir, Abeer Saleh Alshaya, Abduelmenem Alashkham

**Affiliations:** 1 Edinburgh Medical School, Department of Anatomy, University of Edinburgh, Edinburgh, United Kingdom; 2 Department of Anatomy, College of Medicine, King Saud bin Abdulaziz University for Health Sciences, Riyadh, Saudi Arabia; 3 Department of Anatomy, Faculty of Medicine, Universiti Kebangsaan Malaysia, Kuala Lumpur, Malaysia; 4 Public Authority for Applied Education and Training (PAAET), Kuwait City, Kuwait; 5 Zawia Faculty of Medicine, University of Zawia, Zawia, Libya

**Keywords:** abdominal aorta, anatomical variations, cadaver, coeliac trunk, inferior phrenic artery, vascular anatomy

## Abstract

**Introduction:**

The bilateral inferior phrenic arteries supply the diaphragm, the suprarenal glands and the abdominal oesophagus. They typically originate from the abdominal aorta, superior to the coeliac trunk origin, and ascend to the inferior surface of the diaphragm. Variations in their anatomy are common and may contribute to hepatocellular carcinomas’ vascularisation or increase the risk of iatrogenic injury during abdominal procedures. Existing studies are geographically limited and restricted to non-European populations. To date, no published data describing inferior phrenic arterial variations within a Scottish population. This study aims to investigate the origin, course and branching patterns of the inferior phrenic arteries in Scottish cadavers.

**Methods:**

Six formalin-embalmed human cadavers (mean age 87.3 years) from the Anatomy, Edinburgh Medical School, regulated by the Human Tissue (Scotland) Act 2006. The abdominal region was dissected to reveal the abdominal aorta and its branches. The origin, course and branches of the left and right inferior phrenic arteries were observed and documented.

**Results:**

Bilateral inferior phrenic arteries from two female donors (*n* = 4, 33.33%) originated from the coeliac trunk. The inferior phrenic arteries ran anterosuperiorly and laterally to supply the suprarenal glands and the inferior surface of the diaphragm. The remaining eight (66.67%) inferior phrenic arteries emerged from the abdominal aorta bilaterally.

**Conclusion:**

Inferior phrenic arteries with a coeliac trunk origin were found in a third of the Scottish cadaver samples. Knowledge of these variations is crucial to minimising incomplete hepatocellular carcinoma embolization and iatrogenic injuries during upper abdominal surgeries, possibly resulting in haemothorax.

## Introduction

The inferior phrenic arteries predominantly supply the respiratory diaphragm and suprarenal glands (1), with recognised accessory supply to lung metastases (2) and hepatocellular carcinomas (3). Hence, proper identification of the inferior phrenic arteries is pivotal in optimising outcomes in diagnostic radiography, including adrenal arteriography (4), and interventional radiography, particularly during transarterial chemoembolisation of pulmonary and hepatocellular carcinomas (2, 5). Additionally, a comprehensive assessment of the inferior phrenic arterial anatomy is essential to minimise the risk of iatrogenic vascular injury during upper abdominal surgeries, such as laparoscopic gastrectomy (6).

In a classical anatomical presentation, the bilateral inferior phrenic arteries arise from the abdominal aorta, inferior to the aortic hiatus of the diaphragm and superior to the coeliac trunk origin (1). Each artery typically courses anterior to the respective crus of the diaphragm, ascending laterally before dividing into ascending (anterior) and descending (posterior) branches that supply the diaphragm. Along their course, additional branches may arise, including superior suprarenal, diaphragmatic hiatal and accessory splenic arteries (1, 7).

Nevertheless, anatomical variations in the origins and branching patterns of the inferior phrenic arteries have been noted in the literature (8–19). Regarding the variant origins, the common trunk origin of the bilateral arteries has a pooled prevalence of 24.2% compared to independent origins in a global meta-analysis study (9). Variant origins of the inferior phrenic arteries have been reported, with the major variants being the abdominal aorta and the coeliac trunk, followed by renal arteries and left gastric arteries, with rare cases including common hepatic artery, hepatic artery proper, superior mesenteric artery, and left renal artery (9, 20, 21). Rarely, the inferior phrenic artery exhibited a common trunk origin with other arteries, including the left gastric, superior suprarenal, middle suprarenal, inferior suprarenal, and accessory renal arteries (11–14, 22, 23).

Substantial variability has also been observed in the branching patterns of inferior phrenic arteries, with accessory splenic branches arising from the left inferior phrenic artery in 12% of cases in a cadaveric study (7). Although gastric branches most commonly derive from the left inferior phrenic artery, an infrequent origin from the right counterpart has been documented (8). Furthermore, the middle suprarenal branches may arise bilaterally from the inferior phrenic arteries (7). Seldom, these arteries supply aberrant branches to sequestered lung segments, resulting in recurrent haemoptysis (24), or contribute to the vascularisation of lung metastases, especially in the lower lung lobes (2).

Given the significant clinical implications in diagnostic and interventional procedures, a comprehensive understanding of the inferior phrenic arterial anatomy is essential. Nevertheless, the literature thus far covers limited geographical regions, mainly focusing on the United States of America, Japan, Turkey and India (9). To date, no investigation has been conducted on the anatomical variants of the inferior phrenic arteries within the Scottish population. Therefore, this exploratory descriptive study aimed to investigate the origin, course and branching patterns of the inferior phrenic arteries in a Scottish population sample using a cadaveric approach. The purpose was to better comprehend the prevalence of inferior phrenic arterial variants in this population, contributing to the limited literature and offering anatomical insights relevant to surgeons and radiologists.

## Methods

Specimens in this study were obtained from the Department of Anatomy, University of Edinburgh, regulated by the Human Tissue (Scotland) Act 2006. Ethical approval number ANATED_ 0037 was given for the use of cadaveric images.

The upper abdominal regions of six Genelyn-fixed human cadavers (two males and four females) with a mean age of 87.3 years were dissected, totalling 12 inferior phrenic arteries. Genelyn is a proprietary embalming solution kit generally containing 5%–10% formaldehyde and other ingredients, including methanol and phenol, commonly used in cadaver preservation for surgical skill training and medical education (25). Compared to Thiel's embalming fluid, Genelyn is more cost-effective and convenient while still offering life-like tissue colour, texture and pliability to maximise anatomical accuracy (25); it was therefore selected for cadaver fixation in the departmental protocol. A midline abdominal incision with bilateral extensions was made, followed by the removal of abdominal organs to expose the retroperitoneal structures. The dissection was performed to achieve full exposure of the abdominal aorta and its branches. The abdominal aorta and coeliac trunk were carefully identified by removing surrounding peritoneal and adipose tissues. The inferior phrenic arteries were identified, arising either from the aorta superior to the coeliac trunk or directly from the coeliac trunk (26). Their origin, course and branches of the left and right inferior phrenic arteries were observed, documented and photographed.

## Results

**Figure 1:**
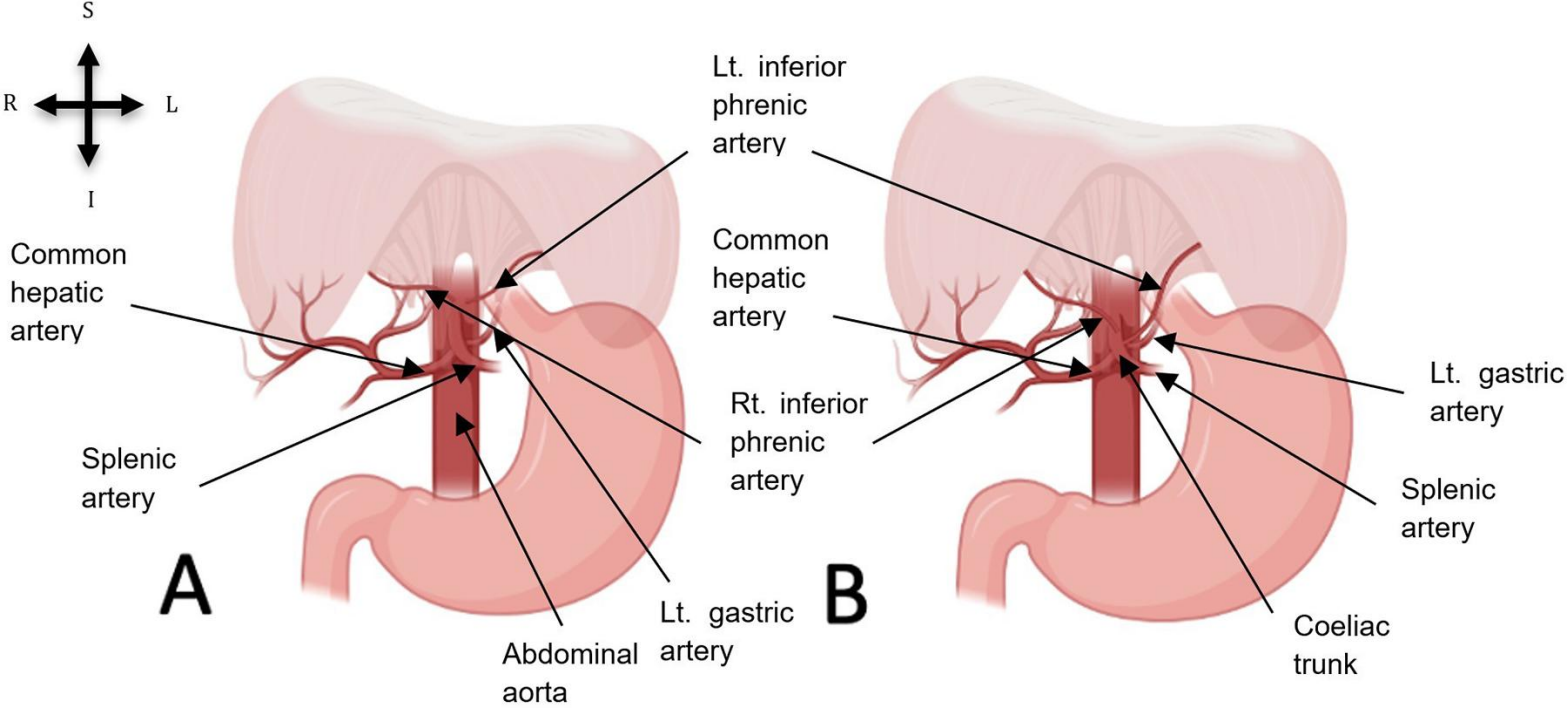
All observed inferior phrenic artery presentations in this study: **(A)** normal origin of bilateral inferior phrenic arteries from the abdominal aorta. **(B)** Coeliac trunk origin of bilateral inferior phrenic arteries. Created with BioRender.com.

**Figure 2:**
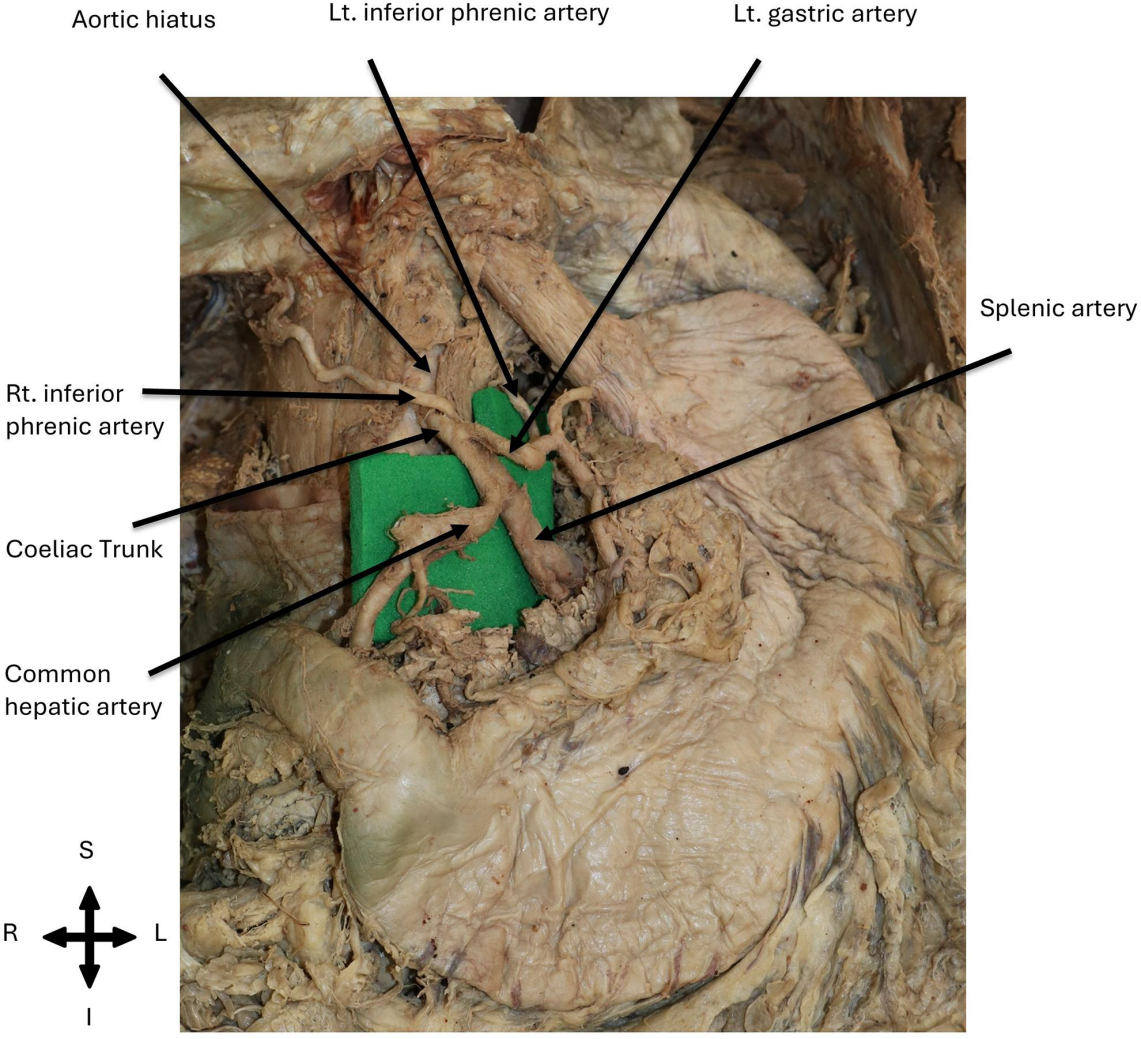
Cadaveric dissection showing the coeliac trunk origin variation observed in one female body donor. The coeliac trunk gave rise to the bilateral inferior phrenic arteries, which emerged independently from the origin, coursed anterosuperiorly and laterally towards the inferior surface of the diaphragm. Two female body donors in total presented such variation in this study.

The origin of the inferior phrenic arteries revealed anatomical variations among the examined cadavers. Out of the 12 inferior phrenic arteries, four (33.33%) were found to originate bilaterally and independently from the coeliac trunk in two female cadavers. The common hepatic, left gastric and splenic arteries, typically emerging from the coeliac trunk, were also observed. An illustration of this coeliac trunk origin (independent origin) variation of the bilateral inferior phrenic arteries is provided in Figure 1(B) while the presentation of this variation in the two female donors was exemplified in Figure 2. The bilateral arteries had a classic course, running anterosuperiorly and laterally to the abdominal surface of the diaphragm, as shown in Figure 2.

The remaining 8 (66.67%) inferior phrenic arteries were found to originate from the abdominal aorta bilaterally and independently, following their traditional course and branching pattern as illustrated in Figure 1(A). The course and branches of the inferior phrenic arteries also had the typical presentation. The bilateral arteries ascended obliquely and laterally to the abdominal surface of the diaphragm, giving off superior suprarenal branches to the ipsilateral adrenal glands.

## Discussion

This exploratory observational study is the first experiment targeting the inferior phrenic arteries and their anatomical variations within a Scottish cadaveric population, addressing a critical gap in regional anatomical data and highlighting the clinical importance of recognising these variations. The findings indicated that one-third (33.33%) of the specimens had bilateral inferior phrenic arteries originating independently from the coeliac trunk, while the remaining 66.67% arose independently from the abdominal aorta. Despite the small sample size, the study provides novel insights into the presence of variant inferior phrenic artery origins in the Scottish population, which can be further examined in a larger cohort to increase the representability of the results.

The coeliac trunk origin variant is described as type A by Loukas et al. (7) or type 2 by Szewczyk et al. (27). The current study results are slightly lower than a previous pooled global prevalence of 46.1% and 35.7% for the respective left and right inferior phrenic arteries having the coeliac trunk origin (9). In contrast with prior studies (14, 24), rare inferior phrenic artery variant origins, such as from the renal arteries or left gastric artery, were not observed, likely due to the small sample size. The course and branching patterns of the inferior phrenic arteries in this study demonstrated the classic presentation, with variant branches, including gastric, middle suprarenal and accessory splenic branches (7, 8), being absent.

The current study results were consistent with prior records of the inferior phrenic artery having a coeliac trunk origin variation in different populations, albeit with population-specific disparities. The coeliac trunk origin variant without a common stem has a 33.33% prevalence in this study, within the prevalence range of 2.2 to 68.6% across different studies (7, 10, 20, 21, 27–34). Interestingly, lower prevalence was observed in Asia, for example, 8.7% and 2.2% for left and right inferior phrenic arteries respectively, in an Indian population (29), compared to Europe, where prevalence ranged from 51.2% to 68.6% in a Czech sample (34). The possible ethnic influences on anatomical variations underpin the importance of investigating the variant inferior phrenic artery anatomy in different populations, which can offer more tailored anatomical insights for the local surgical teams regarding differential risk profiles of such anatomical variations in their population.

Notably, all observed variations in this study were found exclusively in female cadavers, which contradicts prior reports of a higher frequency in males (27, 35). However, multiple studies did not mention the sex-specific prevalences of the inferior phrenic artery variations (20, 29, 34, 36), hindering possible inter-study comparisons. Despite sex differences being acknowledged in general foetal growth patterns (37), the reasons contributing to the sex-specific prevalences of such artery variants remain unclear. Investigation into potential sex-specific genetic or hormonal influences on artery growth patterns could also be explored to clarify the sex-dependent inferior phrenic artery variations.

Differences in embryological development patterns, especially regarding the differential growth rates of abdominal arteries (38), may contribute to the observed coeliac trunk origin variation of the inferior phrenic arteries. Piao et al. (31) suggested that the inferior phrenic arteries are originally part of an upper abdominal arterial meshwork, anastomosing with various arteries, including the coeliac trunk. Therefore, the coeliac trunk origin variant may be an embryological remnant. The disparate developmental speeds of vessels may also contribute to this variation (31), with the inferior phrenic arteries being secondarily transposed to the coeliac trunk as a result (38). Although studies exploring the direct relationships between genetic factors and inferior phrenic artery variations are absent, vascular formation during embryonic development has been characterised as a highly dynamic process with constant remodelling, influenced by various signalling pathways and the extracellular matrix (39). Further research is warranted to ascertain the direct link between the interplay of genetic and epigenetic factors and the inferior phrenic artery anatomy.

Knowledge of the inferior phrenic artery variations can facilitate the diagnosis of relevant pathologies in the upper abdomen and improve surgical treatment outcomes. In oncology, the right inferior phrenic artery is a key extra-hepatic collateral artery supplying hepatocellular carcinomas, providing collateral vascularisation especially to the tumours in segments I, VII and the bare area of the liver (3, 7), and has been reported to emerge from the coeliac trunk in 41% of hepatocellular carcinoma cases (20). Bilateral inferior phrenic arteries have also been noted to supply lung metastases in 9.2% of patients (2). Hence, the accurate angiographic identification of the inferior phrenic artery origin, including its variants, is paramount to ensure total chemoembolisation (3). In diagnostic radiography, contrast injection into the inferior phrenic arteries is performed as part of the super-selective angiography for examination of the adrenal lesions (4).

Furthermore, recognition of coeliac trunk origin variants of the inferior phrenic arteries and their commonly associated comorbidities is essential to prevent iatrogenic artery injury, given prior studies of variant coeliac trunk structures possibly affecting upper abdominal pathologies and surgeries, including gastric cancer (40), hepatic and renal transplantation (41), hepato-biliary and pancreatic surgeries (42). Iatrogenic inferior phrenic artery injury may be severe, causing haemothorax and warranting transcatheter arterial embolisation (43). Rarely, abdominal surgeries such as laparoscopic gastrectomy can result in inferior phrenic artery injury, with complications including pseudoaneurysm that can be fatal if ruptured (6).

A major strength of the current study's methodology is the adoption of Genelyn-embalmed human cadavers to study anatomical variations. With its cost-efficiency and easy handling of specimens (25), this fixative offers a straightforward fixation procedure, thereby facilitating accurate replication of the protocol and enabling verification or extension of the present findings. The life-like gross anatomical presentation offered by the embalmed cadavers, with high repeatability despite possibly differing post-mortem embalming intervals (44), underscores its suitability in anatomical variation investigation and surgical education, filling a research gap in the current literature with abundant inferior phrenic artery variation studies using CT angiography (10, 20, 45). The current exploratory study has several limitations, including the small sample size (*n* = 12). The findings should therefore be interpreted carefully and not be generalised. Moreover, the lack of comprehensive medical histories of the donors has prevented correlation analysis on the relationship between the inferior phrenic artery variants and ante-mortem comorbidities. Notwithstanding, the study presented is the first cadaveric research in the Scottish population presenting incidence data and morphological observation of inferior phrenic artery variations, enriching contemporary anatomical literature with potential clinical relevance. Given the promising findings from the current study, future investigation with a larger sample size and more diverse samples is warranted to verify the external validity of these results and definitively establish the prevalence of different inferior phrenic artery variations in this population.

In conclusion, this exploratory study found that in one-third of the Scottish cadaveric samples, the inferior phrenic arteries originated from the coeliac trunk. Understanding this anatomical variation, which was not investigated thus far in this population, is crucial to improving radiological diagnosis, facilitating hepatocellular carcinoma chemoembolisation and preventing iatrogenic injury during abdominal surgery. This exploratory study is limited by its small sample size and the absence of detailed medical histories. To further explore the clinical significance of these variations, future research should be conducted on a larger scale and incorporate comprehensive patient histories to assess potential correlations between inferior phrenic artery variants and underlying medical conditions.

## Data Availability

The original contributions presented in the study are included in the article/Supplementary Material, further inquiries can be directed to the corresponding author/s.
