# Correction: Coeliac trunk origin of bilateral inferior phrenic arteries

**DOI:** 10.3389/fsurg.2026.1880410

**Published:** 2026-06-02

**Authors:** Raghad Abdulaziz Almansour, Sumar Chan, Jun Mun Teoh, Rasyidah Rehir, Abeer Saleh Alshaya, Abduelmenem Alashkham

**Affiliations:** 1 Edinburgh Medical School, Department of Anatomy, University of Edinburgh, Edinburgh, United Kingdom; 2 Department of Anatomy, College of Medicine, King Saud bin Abdulaziz University for Health Sciences, Riyadh, Saudi Arabia; 3 Department of Anatomy, Faculty of Medicine, Universiti Kebangsaan Malaysia, Kuala Lumpur, Malaysia; 4 Public Authority for Applied Education and Training (PAAET), Kuwait City, Kuwait; 5 Zawia Faculty of Medicine, University of Zawia, Zawia, Libya

**Keywords:** abdominal aorta, anatomical variations, cadaver, coeliac trunk, inferior phrenic artery, vascular anatomy

Affiliation “Public Authority for Applied Education and Training (PAAET), Kuwait City, Kuwait” was omitted for author Abeer Saleh Alshaya. This affiliation has now been added for author Abeer Saleh Alshaya.

The original version of this article has been updated

